# The Safety and Efficacy of Routine Administration of Intracameral Vancomycin during Cataract Surgery

**DOI:** 10.1155/2015/813697

**Published:** 2015-11-04

**Authors:** Sloan W. Rush, Duy Vu, Ryan B. Rush

**Affiliations:** ^1^Panhandle Eye Group, 7400 Fleming Avenue, Amarillo, TX 79106, USA; ^2^Texas Tech University Health Sciences Center, 1400 S. Coulter, Amarillo, TX 79106, USA; ^3^Southwest Retina Specialists, 7411 Wallace Boulevard, Amarillo, TX 79106, USA

## Abstract

*Purpose*. To evaluate the safety and efficacy of intracameral vancomycin during cataract surgery using a standardized dosage and delivery technique. *Methods*. The charts of 20,719 consecutive eyes that underwent phacoemulsification with intraocular lens implantation in a single ambulatory surgery center were retrospectively reviewed over a 5-year period. *Results*. The first 11,333 consecutive cases did not receive intracameral vancomycin, whereas the next 9,386 consecutive cases all received intracameral vancomycin. There were no significant differences in the baseline characteristics between the cohort of subjects who received intracameral vancomycin and the cohort of subjects that did not. There were a total of 11 subjects (0.97 cases per 1,000) that developed postoperative endophthalmitis in the group that did not receive intracameral vancomycin, whereas there were no cases of postoperative endophthalmitis in the group that received intracameral vancomycin (*p* = 0.0015). The overall rate of intraoperative and postoperative complications and the final postoperative visual acuities were similar among cohorts. There were no cases of toxic anterior segment syndrome occurring in either group during the study period. *Conclusions*. Routine administration of intracameral vancomycin during cataract surgery significantly decreased the incidence of postoperative endophthalmitis and was not associated with an increased incidence of postoperative adverse events.

## 1. Introduction

Endophthalmitis is a rare but potentially devastating occurrence following cataract surgery [1]. The reported incidence of postoperative endophthalmitis after cataract surgery varies from 1 in 300 to 1 in 14,000 depending upon the geographic location and surgical techniques used [2, 3]. Besides sterile technique, other proven innovations that have decreased the incidence of endophthalmitis following cataract surgery include use of preoperative povidone/iodine cleansing solution and small incision phacoemulsification techniques [4, 5]. At the present time, there is no general consensus in regard to the effectiveness of topical antibiotics for reducing the rates of endophthalmitis after cataract surgery [6].

The use of intracameral antibiotics during cataract surgery has been reported since the 1990's [7]. Since that time, practitioners have experimented with many different intracameral dosages and drugs, most notably cefuroxime, moxifloxacin, and vancomycin [8–10]. The European cataract surgery trials reported a 5-fold lower incidence in acute postoperative endophthalmitis with intracameral cefuroxime [11]. More recently, a study group from Kaiser Permanente reported between a 2-fold and more than a 10-fold reduction in postoperative endophthalmitis, depending upon which intracameral antibiotic and which dosage were used [12]. Despite these reports, there has been some general resistance among cataract surgeons in regard to the overall implementation of this infection prevention technique either in place of or in combination with topical antibiotic use [13].

In this study, we report the outcomes of subjects from a single ambulatory surgery center before and after implementation of a standardized intracameral vancomycin dosage and delivery method during cataract surgery, especially in regard to the rates of postoperative endophthalmitis and toxic anterior segment syndrome (TASS) or other severe adverse events related to drug toxicity.

## 2. Methods

The SRS Institutional Review Board (IORG0007600/IRB00009122) approved this retrospective, consecutive chart review from January 2010 through June 2015 of all patients that underwent phacoemulsification with intraocular lens (IOL) implantation at a single ambulatory surgery center in Amarillo, TX, USA. All research components adhered to the tenets of the Declaration of Helsinki and were conducted in agreement with human research regulations and standards.

### 2.1. Inclusion/Exclusion Criteria, Data Collection, and Follow-Up

The operative eyes of all patients that underwent phacoemulsification with IOL implantation on the Centurion Vision System (Alcon, Fort Worth, TX, USA) by one of four surgeons during the aforementioned study interval were included. The baseline characteristics, intraoperative details, and postoperative outcomes were collected. The baseline characteristics included subject age, gender, preoperative best spectacle corrected visual acuity (BSCVA), axial length measured on the Zeiss IOL Master (Carl Zeiss Meditec AG, Jena, Germany), average keratometry measured on the Zeiss IOL Master, existing ocular comorbidities, and history of previous ocular surgery. The intraoperative data collected from each case included whether or not the patient received intracameral vancomycin, the development of a posterior capsular tear with vitreous prolapse, the dislocation of lens fragments into the posterior segment, the performance of an anterior vitrectomy, and whether or not the patient's surgery was billed as a complex procedure. Complex procedures included but were not limited to the intraoperative use of trypan blue (Vision Blue, Dutch Ophthalmic USA, Exeter, NH), pupillary expansion techniques of any kind such as sphincterotomies, iris hooks or Malyugin ring (MicroSurgical Technology, Redmond, WA) placement, lysis of anterior synechiae, or placement of a capsular tension ring. The postoperative outcomes included BSCVA at 3 months, the occurrence of postoperative endophthalmitis, the development of TASS, or any other unexplained corneal edema and/or excessive intraocular inflammation and whether or not the patient had occurrence of any other complications during the postoperative period.

### 2.2. Pre-, Peri-, and Intraoperative Routine

Preoperative antibiotics were not given to any patient during the study interval. All patients received iodine/povidone surgical scrub prior to draping using the following standardized 3-minute scrub technique: 30 mL of povidone-iodine 5% solution was placed onto a sterile tray. Using sterile gloves, multiple drops of the 5% solution were placed onto the ocular surface. Sterile cotton tipped applicators were used to scrub the solution along the eyelids, eyelashes, and the inferior ocular fornix. Then sterile 4 × 4 gauze soaked in the 5% solution was used to scrub the periocular skin in an expanding circular fashion. The povidone-iodine solution was flushed off of the ocular surface and the periocular skin using sterile balanced salt solution. Finally, the skin was dried using sterile 4 × 4 gauze. This technique remained constant among all subjects included in the study.

All surgeons made clear corneal incisions with 2.4 mm keratomes and 1 mm side port incisions. There were no scleral tunnel incisions performed during the study. Phacoemulsification techniques varied according to surgeon preference. None of the surgeons used antibiotics in the infusion fluid. Corneal wound suturing was at the discretion of the surgeon but in general was rarely performed.

### 2.3. Intracameral Injection Technique

The intracameral vancomycin was obtained from the same local compounding pharmacy in all instances. It was prepared in the following manner: vancomycin solution was diluted to 1% using normal saline in a USP 797 clean room under an ISO Class 5 hood. Then 0.2 mL of the solution was drawn up into a TB syringe and capped. For the cohort that received intracameral vancomycin, the following standardized technique was used: the preloaded syringe was dropped onto the sterile field as the last step of the cataract procedure once the wounds were tested and found to be sealed. The vancomycin syringe was uncapped and placed on a 30-gauge blunt irrigating cannula. After irrigating air bubbles out of the syringe, the cannula was placed into the 1 mm side port incision and 0.1 mL of vancomycin 1% solution (or 1 mg total) was injected under the anterior capsule and into the capsular bag. The cannula was withdrawn while gently injecting fluid on the way out to maintain appropriate inflation of the anterior chamber. No further wound hydration or manipulation was performed.

### 2.4. Postoperative Routine

Patients that received retrobulbar blocks were given neomycin/polymyxin B/dexamethasone ophthalmic ointment (Bausch & Lomb Inc., Tampa, FL) prior to patching of the operative eye. Patients that had topical anesthesia received one drop of generic prednisolone acetate 1% and one drop of generic ofloxacin 0.3% prior to placement of a clear plastic shield. Each surgeon used Besivance (Bausch & Lomb Inc, Tampa, FL) three times daily for 2 weeks total on all patients after surgery regardless of whether or not they received intracameral vancomycin. Each surgeon used varying doses and frequencies of topical corticosteroids and topical nonsteroidal anti-inflammatory drugs during the postoperative period.

### 2.5. Statistical Analysis

The JMP 11 mathematical software package from the SAS Institute (Cary, NC, USA) was used to perform the statistical analysis and calculate means with standard deviations. Since the outcome variables are not assumed to have a normal distribution, one-way analysis of the variance (and likelihood ratios, when appropriate for nominal variables) was used to compare the baseline characteristics, intraoperative details, and postoperative outcomes among the group that did not receive intracameral vancomycin and the group that received intracameral vancomycin. Since the study population is relatively small compared to the reported incidence of endophthalmitis, two-tailed Fisher's exact test was used when comparing these distributions. Results were considered statistically significant at alpha <0.05 level.

## 3. Results

A total of 20,719 consecutive cataract surgeries were included in the analysis. The first 11,333 surgeries did not receive intracameral vancomycin, whereas the next 9,386 all received intracameral vancomycin. None of the subjects in the study had intracameral vancomycin withheld because of an allergy. The means and distributions of the baseline characteristics for each group are compared in Table 1. There was no statistical difference among these two cohorts as it relates to preoperative characteristics.  Table 2 compares the intraoperative details and postoperative outcomes among the two groups. The group not receiving intracameral vancomycin had more than 10 times the likelihood of developing postoperative endophthalmitis during the study interval (*p* = 0.0015), whereas there were no other identifiable outcome differences among the two groups. Notably, there were no cases of TASS or other unexplained occurrences of persistent corneal edema or prolonged anterior chamber inflammation in the intracameral vancomycin group to suggest the possibility of an adverse reaction related to medication toxicity.

Table 3 summarizes the characteristics and outcomes of the 11 cases of endophthalmitis that occurred in the group that did not receive intracameral vancomycin. All subjects presenting with signs and symptoms of postoperative infectious endophthalmitis received 1-mg of intravitreal vancomycin and 2.25-mg of intravitreal ceftazidime promptly by a retina specialist. Cultures were not routinely taken, so this data is not available.

## 4. Discussion 

The incidence of postoperative endophthalmitis significantly decreased after intracameral vancomycin was routinely implemented using a standardized technique in our study population. The incidence of endophthalmitis in our study is comparable to other large series that have studied intracameral vancomycin administration during cataract surgery [10, 14]. Specifically, in order to prevent one case of endophthalmitis, 1,030 cataract surgery patients needed to prophylactically receive intracameral vancomycin during our study.

Safety concerns related to intracameral drug toxicity and the lack of availability of an FDA-approved product in the United States with appropriate sterility, manufacturing, and chemistry controls have often been cited as the main reasons why many cataract surgeons do not routinely use intracameral antibiotics [15–17]. It is noteworthy that we did not have any cases of TASS or unexplained persistent corneal edema and/or intraocular inflammation out-of-proportion to what was expected early in the postoperative period in either of our cohorts. Witkin et al. recently reported a case series of hemorrhagic occlusive retinal vasculitis that occurred within two weeks after routine uncomplicated cataract surgery [18]. These authors described this complication as possibly being related to use of intracameral vancomycin during the cataract surgery. In our series, we did not have any cases of hemorrhagic occlusive retinal vasculitis to report in either of our cohorts. While both postoperative endophthalmitis and retinal vasculitis can have devastating effects on final visual outcomes, we did not find a link between intracameral vancomycin and retinal vasculitis, and if such an association actually exists, we suspect that the incidence of hemorrhagic occlusive retinal vasculitis is much less than that of postoperative endophthalmitis.

Weaknesses of our study include the retrospective nature of the study design, the lack of a contemporaneous control group, and the possibility of missed cases of endophthalmitis due to lack of follow-up. Future prospective studies are required to fully determine safety and efficacy of the technique described in this study recounting the routine use of intracameral vancomycin for the prevention of postoperative endophthalmitis following cataract surgery. In conclusion, we report that the routine administration of intracameral vancomycin during cataract surgery significantly decreased the incidence of postoperative endophthalmitis and was not associated with an increased incidence of postoperative adverse events related to compounded vancomycin toxicity.

## Figures and Tables

**Table 1:** The means and distributions with standard deviations of the baseline characteristics among the group that received and the group that did not receive intracameral vancomycin during cataract surgery.

Baseline characteristic	Did not receive intracameral vancomycin (*n* = 11,333)	Did receive intracameral vancomycin (*n* = 9,386)	*p* value
Age (years)	71.3 (8.5)	70.6 (9.0)	*p* = 0.5325
Gender	Male = 5,610 (49.5%) Female = 5,723 (50.5%)	Male = 4,623 (49.3%) Female = 4,763 (50.7%)	*p* = 0.7326
Laterality	Right eye = 5,689 (50.2%) Left eye = 5,644 (49.8%)	Right eye = 4,698 (50.1%) Left eye = 4,688 (49.9%)	*p* = 0.3658
Preoperative BSCVA (logMAR)	0.51 (0.26)	0.49 (0.31)	*p* = 0.5099
Axial length (mm)	23.9 (1.5)	24.1 (1.4)	*p* = 0.3657
Average keratometry (diopters)	43.2 (2.2)	43.3 (2.3)	*p* = 0.8523
Underlying ocular Comorbidities	Yes = 1,190 (10.5%) No = 10,143 (89.5%)	Yes = 975 (10.4%) No = 8,411 (89.6%)	*p* = 0.7921
Previous history of ocular surgery	Yes = 1,473 (13.0%) No = 9,860 (87.0%)	Yes = 1,239 (13.2%) No = 8,147 (86.8%)	*p* = 0.6662

**Table 2:** The means and distributions with standard deviations of the intraoperative and postoperative outcomes among the group that received and the group that did not receive intracameral vancomycin during cataract surgery.

Outcome measure	Did not receive intracameral vancomycin (*n* = 11,333)	Did receive intracameral vancomycin (*n* = 9,386)	*p* value
Posterior capsular tear	Yes = 56 (0.5%) No = 11,277 (99.50%)	Yes = 49 (0.53%) No = 9,337 (99.47%)	*p* = 0.8752
Anterior vitrectomy performed	Yes = 44 (0.39%) No = 11,289 (99.61%)	Yes = 35 (0.37%) No = 9,351 (99.63%)	*p* = 0.8583
Dislocation of lens fragments into the posterior segment	Yes = 14 (0.13%) No = 11,319 (99.87%)	Yes = 9 (0.10%) No = 9,377 (99.90%)	*p* = 0.8463
Complex procedure billed	Yes = 985 (8.7%) No = 10,348 (91.3%)	Yes = 808 (8.6%) No = 8,578 (91.4%)	*p* = 0.8488
Postoperative BSCVA (logMAR)	0.15 (0.15)	0.13 (0.14)	*p* = 0.1896
Postoperative endophthalmitis	Yes = 11 (0.09%) No = 11,322 (99.9%)	Yes = 0 (0%) No =9,386 (100%)	*p* = 0.0015

**Table 3:** Summary of the characteristics and outcomes of subjects developing postoperative endophthalmitis in the cohort that did not receive intracameral vancomycin.

Case ID	Age/gender	Complications during the cataract surgery	Complex cataract surgery billed	Previous ocular surgery	Underlying ocular comorbidities	Presentation of intraocular infection symptoms to clinician after cataract surgery	Presenting BSCVA (Snellen)	Eventual pars plana vitrectomy performed	Final BSCVA (Snellen)

1	72-year-old male	No	No	No	No	7 days	20/200	No	20/20

2	64-year-old female	No	No	Yes Eight incision radial keratotomy	No	4 days	20/400	No	20/20

3	74-year-old female	No	No	No	No	36 days	20/200	Yes	20/20

4	69-year-old male	No	Yes Trypan blue	Yes Penetrating keratoplasty for pellucid marginal degeneration	Yes Corneal transplant	27 days	Hand motions	Yes	20/25

5	54-year-old male	No	Yes Trypan blue	Yes Penetrating keratoplasty for keratoconus	Yes Corneal transplant	21 days	20/800	No	20/25

6	81-year-old male	No	No	No	No	3 days	Hand motions	No	20/30

7	75-year-old male	No	No	No	No	4 days	Hand motions	No	20/40

8	69-year-old male	No	No	No	No	5 days	20/400	No	20/20

9	78-year-old female	No	Yes Pupillary expansion with sphincterotomies	No	No	7 days	20/400	No	20/25

10	71-year-old female	No	No	No	Yes Nonproliferative diabetic retinopathy	12 days	Hand motions	No	20/25

11	80-year-old female	No	No	No	No	3 days	20/80	No	20/20
